# Extracellular Vesicle from *Chlorella vulgaris* Alleviates Hepatic Fibrosis in a Mouse Model of Metabolic Dysfunction-Associated Steatotic Liver Disease Through Modulation of Inflammatory Signaling

**DOI:** 10.3390/ijms27093735

**Published:** 2026-04-22

**Authors:** Hinata Harada, Yusuke Ohsaki, Afifah Zahra Agista, Hsin-Jung Ho, Takuo Hirose, Kotaro Yamada, Mutsumi Furukawa, Tomonori Nochi, Wan-Chun Chiu, Ya-Ling Chen, Chiu-Li Yeh, Suh-Ching Yang, Takefumi Mori, Hitoshi Shirakawa

**Affiliations:** 1 Laboratory of Nutrition, Graduate School of Agricultural Science, Tohoku University, Sendai 980-8572, Japanagista@g-mail.tohoku-university.jp (A.Z.A.); hjho@tohoku.ac.jp (H.-J.H.); shirakah@tohoku.ac.jp (H.S.); 2 International Education and Research Center for Food Agricultural Immunology, Graduate School of Agricultural Science, Tohoku University, Sendai 980-8572, Japan; mutsumi.furukawa.c4@tohoku.ac.jp (M.F.); nochi@tohoku.ac.jp (T.N.); 3 Institute for Excellence in Higher Education, Tohoku University, Sendai 980-8576, Japan; 4 Division of Physiology, Faculty of Medicine, Tohoku Medical and Pharmaceutical University, Sendai 983-8536, Japan; hirose.takuo@tohoku-mpu.ac.jp; 5 Division of Nephrology and Hypertension, Faculty of Medicine, Tohoku Medical and Pharmaceutical University, Sendai 983-8536, Japan; 6 Division of Integrative Renal Replacement Therapy, Faculty of Medicine, Tohoku Medical and Pharmaceutical University, Sendai 983-8536, Japan; 7 Regulatory Affairs Promotion Division, ROHTO Pharmaceutical Co., Ltd., Osaka 544-8666, Japan; yamadakotaro@rohto.co.jp; 8 Laboratory of Animal Functional Morphology, Graduate School of Agricultural Science, Tohoku University, Sendai 980-8572, Japan; 9 School of Nutrition and Health Sciences, College of Nutrition, Taipei Medical University, Taipei 11031, Taiwan; wanchun@tmu.edu.tw (W.-C.C.); ylchen01@tmu.edu.tw (Y.-L.C.); clyeh@tmu.edu.tw (C.-L.Y.); sokei@tmu.edu.tw (S.-C.Y.)

**Keywords:** MASLD, microalgae, *Chlorella vulgaris*, liver disease, extracellular vesicles, exosome, hepatic steatosis, hepatic fibrosis, inflammation, macrophage, Kupffer cell

## Abstract

Metabolic-dysfunction-associated steatotic liver disease (MASLD) is a major chronic liver disorder that progresses through inflammation and fibrosis to cirrhosis, yet no effective pharmacological therapy is available. Extracellular vesicles (EVs), which are key mediators of intercellular communication, have recently been reported to exert preventative and therapeutic effects in disease models. This study evaluated the oral efficacy of EVs derived from the microalga *Chlorella vulgaris* (CEVs) in an MASLD mouse model. Male C57BL/6J mice were assigned to a control group (normal diet), an MASLD group (choline- and methionine-deficient high-fat diet; CDHF), or CEV group (CDHF + CEVs). Twelve-week CEV administration did not alter the CDHF-induced reduction in circulating lipid levels or produce an increase in hepatic lipid content. However, CEV treatment significantly suppressed CDHF-induced fibrosis with collagen accumulation and reduced the mRNA expression of fibrosis-related genes, including *Col1a1*, *Acta2*, *Mmp2*, and *Timp1*. CEVs also significantly downregulated the expression of macrophage-derived inflammatory mediators—*Ccl2*, *Ccr2*, *Il6* and *Il1b*—and reduced lobular inflammatory foci. These findings suggest that CEVs attenuate hepatic fibrosis by modulating early inflammation associated with steatosis and inhibiting hepatic stellate cell activation. This study supports the potential of CEVs as a novel oral intervention for slowing MASLD progression.

## 1. Introduction

Metabolic-dysfunction-associated steatotic liver disease (MASLD) is a chronic liver disorder characterized by hepatic steatosis in the absence of excessive alcohol consumption and accompanied by metabolic abnormalities such as obesity and type 2 diabetes [1,2]. The prevalence of MASLD has been rising rapidly in parallel with the global increase in obesity and type 2 diabetes [3]. A meta-analysis of studies conducted between 2016 and 2019 estimated that more than 38% of the global adult population is affected [4], and the prevalence is projected to exceed 55% by 2040 [5]. Excessive hepatic lipid accumulation observed in MASLD triggers inflammation and can progress to fibrosis, termed metabolic-dysfunction-associated steatohepatitis (MASH), ultimately leading to cirrhosis and hepatocellular carcinoma. Although hepatocytes possess a remarkable regenerative capacity, and recent evidence suggests that fibrosis and cirrhosis may be reversible [6], MASLD remains an important risk factor for cardiovascular disease, underscoring the need for prevention and early intervention [7]. Currently, no effective pharmacological therapy for MASLD has been established [8], and lifestyle modifications—including dietary improvement and appropriate physical activity—remain the primary therapeutic strategies [7].

Extracellular vesicles (EVs) have emerged as a promising strategy for the prevention and treatment of disease. EVs are nanosized, lipid bilayer-enclosed vesicles secreted by cells from diverse species, including humans, animals, microorganisms, and plants [9]. They encapsulate bioactive molecules such as proteins, nucleic acids (including mRNAs and miRNAs), and lipids and mediate intercellular communication by transporting these cargos [10]. EVs derived from mammalian cells have been reported to exert various physiological functions, including tissue repair and immunomodulation [11,12]. However, their practical application as functional foods or orally administered therapeutics remains limited due to challenges such as difficulties in large-scale production, potential immunogenicity, and poor permeability across the intestinal barrier [13,14]. In contrast, EVs derived from plants and algae are easier to produce, exhibit high biocompatibility, and show resistance to degradation by intestinal digestive enzymes. Consequently, several studies have demonstrated that orally administered plant- and algae-derived EVs can be stably absorbed in vivo [15,16,17,18], highlighting their potential for development as orally available agents to ameliorate disease conditions.

*Chlorella vulgaris* (*C. vulgaris*) is a unicellular microalga composed of proteins, lipids, dietary fiber, and other nutrients [19]. It contains abundant phytochemicals with anti-inflammatory and antioxidant properties, including chlorophylls, carotenoids, and tocopherols [20]. Several studies have reported that *C. vulgaris* improves glucose metabolism [21] and dyslipidemia [22], and it has long been utilized commercially as a functional food [23]. Moreover, the beneficial effects of *C. vulgaris* on MASLD have been demonstrated in both animal models and human clinical trials [24,25]. Recently, Jin et al. reported that *C. vulgaris*, similar to other microalgae, secretes EVs [26]. These findings raise the possibility that *C. vulgaris*-derived EVs (CEVs) may exert therapeutic effects on liver diseases. However, the impact of orally administered CEVs on MASLD has not been sufficiently investigated.

A variety of approaches, including dietary, genetically engineered, and chemically induced models, have been developed to recapitulate MASLD pathology in preclinical studies [27]. Among these, the choline- and methionine-deficient high-fat diet (CDHF) model can induce hepatic steatosis, inflammation, and fibrosis within a relatively short period compared with other models [28]. Under conditions of choline and methionine deficiency, impaired phosphatidylcholine synthesis leads to defective production of very-low-density lipoproteins (VLDLs), which are required for triacylglycerol (TG) export, resulting in lipid accumulation within hepatocytes [29]. Whereas a high-fat or Western diet often fail to induce fibrosis, even after more than 36 weeks of feeding, choline- and methionine-deficient diets have been reported to induce significant fibrosis after as few as eight weeks [27]. Because human MASLD encompasses a broad pathological spectrum ranging from simple steatosis to steatohepatitis and fibrosis, the CDHF model serves as a valuable preclinical tool for reproducing the fibrotic features of MASLD.

In this study, we investigated the effects of orally administered CEVs on hepatic steatosis and fibrosis in a CDHF-fed MASLD mouse model. CEV administration attenuated collagen deposition within the hepatic interstitium and reduced the mRNA expression of fibrosis- and inflammation-related genes. These findings indicate that CEVs may suppress inflammation that drives fibrogenesis and thereby mitigate the risk of progression to MASH.

## 2. Results

### 2.1. Physicochemical Properties and Bioavailability of C. vulgaris-Derived EVs

**Figure 1:**
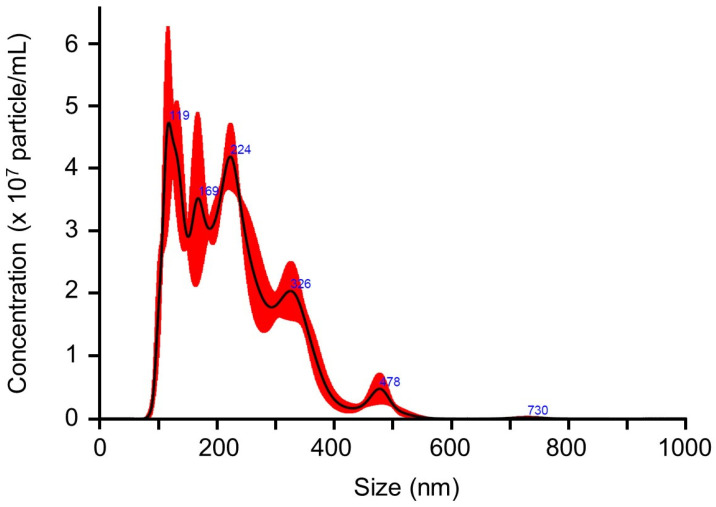
Size distribution of CEV nanoparticles. The data are presented as the mean (black line) ± standard error (red line). CEV, *Chlorella vulgaris*-derived extracellular vesicles.

CEVs were prepared by isolating and concentrating the culture supernatant of *C. vulgaris* using ultrafiltration, followed by resuspension in PBS as described by Morishita et al. [30]. Prior to evaluating their hepatoprotective effects in the MASLD mouse model, their physicochemical and morphological characteristics and bioavailability were assessed by particle size distribution, scanning electron microscopy imaging, nanoparticle concentration, protein profile, and stability after oral administration. Nanoparticle tracking analysis using the NanoSight system revealed that CEVs exhibited a particle size distribution of 100–250 nm, with an average diameter of 227.6 nm (Figure 1). The nanoparticle concentration ranged from 3.26 to 6.89 × 10^10^ particles/mL, and the total protein concentration was 1.12 μg/mL. Scanning electron microscopy further confirmed the presence of spherical vesicles with diameters consistent with the results of nanoparticle tracking analysis (Supplementary Figure S1).

To further characterize the protein components of CEVs, we performed proteinase K digestion in the presence or absence of SDS, followed by SDS-PAGE analysis. Multiple protein bands were detected in untreated samples. Several bands disappeared after proteinase K treatment without SDS, whereas others remained detectable unless SDS was added (Supplementary Figure S2).

To examine whether orally administered CEVs enter systemic circulation, plasma samples collected 1 h and 6 h after CEV administration were analyzed by PCR. *C. vulgaris*-derived DNA was detected only in plasma collected 1 h after administration, indicating that at least a portion of the administered CEVs or their nucleic acid cargo reached the bloodstream (Supplementary Figure S3). No *C. vulgaris*-derived DNA was detected in plasma collected at 6 h.

### 2.2. Effects of CDHF Feeding and CEV Administration on Body Weight and Food Efficiency

**Figure 2:**
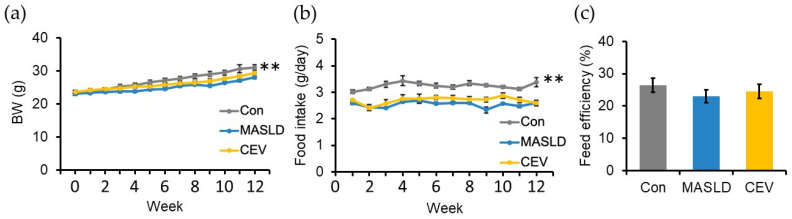
Body weight and food intake in CDHF diet-induced MASLD model mice. (**a**) Body weight over the 12-week experimental period; (**b**) weekly average food intake; (**c**) food efficiency, calculated as the ratio of total body weight gain to total food intake during the experimental period. Con, control; MASLD, metabolic dysfunction-associated steatotic liver disease; BW, body weight; CEV, *Chlorella vulgaris*-derived extracellular vesicles. Data are expressed as mean ± SE (*n* = 8 per group). Statistical analyses were performed using two-way repeated-measures ANOVA followed by Dunnett’s post hoc test for panels (**a**,**b**) and one-way ANOVA followed by Dunnett’s test for panel (**c**); ** *p* < 0.01 vs. MASLD group.

Body weight increased steadily in all groups of male C57BL/6J mice during the 12-week feeding period; however, the increase was significantly smaller in the CDHF-fed MASLD group than in the control group (Figure 2a). CEV administration did not affect body weight. Average food intake throughout the experimental period was significantly lower in the MASLD group than in the control group (Figure 2b), with no difference between the MASLD and CEV groups. Feed efficiency, calculated as total body weight gain relative to total food intake, did not differ between the groups (Figure 2c). These findings indicate that CDHF feeding reduced both body weight gain and food intake without altering feed efficiency. CEV administration did not influence any of these parameters.

### 2.3. CEVs Improve Insulin Sensitivity

**Figure 3:**
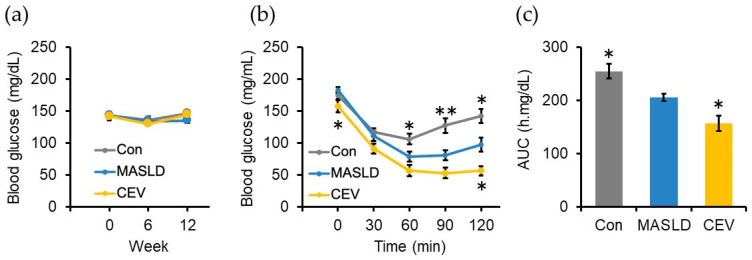
Effect of CEVs administration on insulin sensitivity. (**a**) Non-fasting blood glucose levels at weeks 0, 6, and 12. (**b**) Blood glucose levels during ITT. (**c**) AUC calculated based on blood glucose levels during ITT. Con, control; MASLD, metabolic dysfunction-associated steatotic liver disease; CEV, *Chlorella vulgaris*-derived extracellular vesicles. Data are expressed as mean ± SE (*n* = 8 per group). Statistical analyses were performed using two-way repeated-measures ANOVA followed by Dunnett’s post hoc test for panels (**a**,**b**) and one-way ANOVA followed by Dunnett’s test for panel (**c**); * *p* < 0.05, ** *p* < 0.01 vs. MASLD group.

To assess insulin resistance, a known risk factor for MASLD [1], non-fasting blood glucose levels were measured at weeks 0, 6, and 12. No significant differences were observed between the groups at any time points (Figure 3a). An insulin tolerance test was performed at week 10. Fasting blood glucose levels prior to insulin administration were significantly lower in the CEV group than in the MASLD group. Intraperitoneal insulin injection reduced blood glucose levels in all groups; however, the reduction was significantly greater in the CEV group (Figure 3b). Consistent with this, the area under the curve (AUC) for blood glucose during the test was significantly lower in the CEV group (Figure 3c). These findings suggest that CEV administration ameliorates insulin resistance in MASLD model mice.

### 2.4. CDHF Feeding Increased Plasma Lipid Concentration, While CEV Administration Did Not Affect Plasma Lipid Profiles

To evaluate liver function, plasma levels of hepatic injury markers were measured (Table 1). Plasma alanine aminotransferase (ALT), alkaline phosphatase (ALP), and leucine aminopeptidase (LAP) activities were significantly elevated in the MASLD group compared with the control group; however, CEV administration did not affect these parameters.

Because methionine and choline are required for phosphatidylcholine biosynthesis, a major component of VLDL, CDHF feeding has been reported to impair lipid export from the liver, thereby promoting hepatic lipid accumulation [31]. Consistent with previous findings, plasma levels of TG, total cholesterol (TC), free cholesterol (FC), esterified cholesterol (CE), non-esterified fatty acid (NEFA), low-density lipoprotein cholesterol (LDL-C), and high-density lipoprotein cholesterol (HDL-C) were significantly reduced in the MASLD group. CEV administration did not influence plasma lipid concentrations. Plasma total protein and albumin levels, indicators of hepatic function and nutritional status, did not differ between the groups.

### 2.5. CEV Administration Did Not Affect CDHF-Induced Hepatic Steatosis

**Figure 4:**
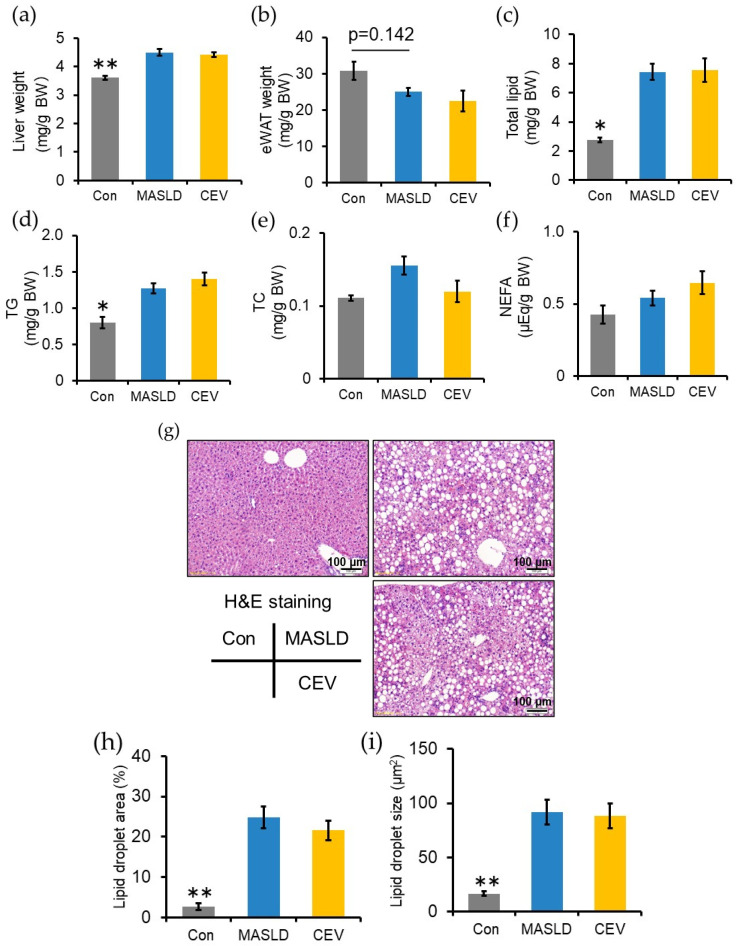
Effect of CEV administration on CDHF-induced hepatic steatosis. (**a**) Relative liver weight and (**b**) epididymal white adipose tissue weight. (**c**–**f**) Hepatic lipid profile; (**c**) total lipid, (**d**) triglyceride (TG), (**e**) total cholesterol (TC), and (**f**) non-esterified fatty acid (NEFA). (**g**) Representative images of hematoxylin and eosin (H&E)-stained liver sections. (**h**) Quantification of hepatic lipid droplet area and (**i**) average droplet size calculated using ImageJ. Con, control; MASLD, metabolic dysfunction-associated steatotic liver disease; CEV, *Chlorella vulgaris*-derived extracellular vesicles; eWAT, epididymal white adipose tissue. Data are expressed as mean ± SE (*n* = 5–8 per group). Statistical analyses were performed using one-way ANOVA followed by Dunnett’s test; * *p* < 0.05, ** *p* < 0.01 vs. MASLD group.

CDHF feeding significantly increased relative liver weight and tended to decrease epididymal white adipose tissue (eWAT) weight, though the difference did not reach statistical significance (Figure 4a,b). Hepatic lipid content was quantified to evaluate steatosis (Figure 4c–f). Total hepatic lipid and TG levels were significantly elevated in the MASLD group compared with the control group, whereas CEV administration did not alter these parameters. Neither CDHF feeding nor CEV treatment affected hepatic TC or NEFA levels.

Histological analysis using hematoxylin and eosin (H&E) staining revealed marked lipid droplet accumulation in both the MASLD and CEV groups (Figure 4g–i). ImageJ software (version 1.37c, Wayne Rasband, National Institute of Health, Bethesda, MD, USA)-based quantification showed that the lipid droplet area (Figure 4h) and mean droplet size (Figure 4i) were significantly higher in the MASLD group than in the control group, with no differences between the MASLD and CEV groups. These results indicate that CDHF feeding induced hepatic lipid accumulation due to impaired lipid export, and CEV administration did not influence CDHF-induced steatosis.

### 2.6. CEVs Suppressed CDHF-Induced Hepatic Fibrosis and Downregulated Fibrosis-Related Gene Expression

**Figure 5:**
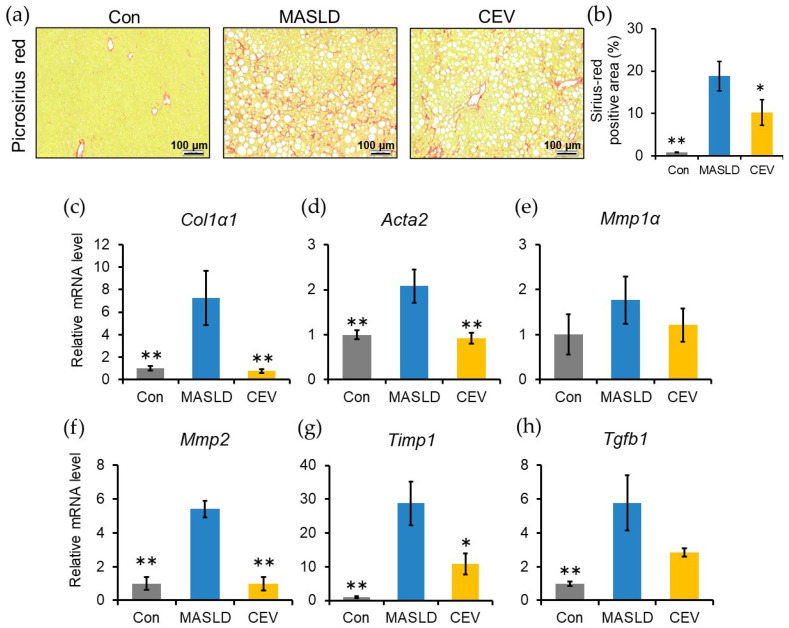
Effect of CEV administration on CDHF-induced hepatic fibrosis. (**a**) Representative images of picrosirius red-stained liver sections. (**b**) Quantification of fibrotic area using ImageJ. (**c**–**h**) Hepatic mRNA expression of fibrosis-related genes: (**c**) *Col1α1*, (**d**) *Acta2* (α-SMA), (**e**) *Mmp1α*, (**f**) *Mmp2*, (**g**) *Timp1*, and (**h**) *Tgfb1*. Con, control; MASLD, metabolic dysfunction-associated steatotic liver disease; CEV, *Chlorella vulgaris*-derived extracellular vesicles. Data are expressed as mean ± SE (*n* = 5–8 per group). Statistical analyses were performed using one-way ANOVA followed by Dunnett’s test; * *p* < 0.05, ** *p* < 0.01 vs. MASLD group.

Fibrosis, a key indicator of hepatic injury in MASLD, was evaluated by picrosirius red staining. Collagen deposition was observed in both the MASLD and CEV groups (Figure 5a). Quantification of the picrosirius red positive area revealed a significant increase in fibrosis in the MASLD group compared with the control group, whereas the CEV group showed a significant reduction relative to the MASLD group (Figure 5b).

Expression of fibrosis-related genes was then examined. Hepatic mRNA levels of type I collagen 1A1 (*Col1α1*), myofibroblast marker α-SMA (*Acta2*), *Mmp2*, and *Timp1* were significantly elevated in the MASLD group compared with the control group, but significantly lower in the CEV group (Figure 5c–g). *Tgfb1* expression level was also significantly increased in the MASLD group (Figure 5h). These findings indicate that CDHF feeding induced hepatic fibrosis and that CEV administration attenuated fibrosis progression.

### 2.7. CEVs Downregulated Inflammatory Gene Expression and Suppressed Immune Cell Infiltration

**Figure 6:**
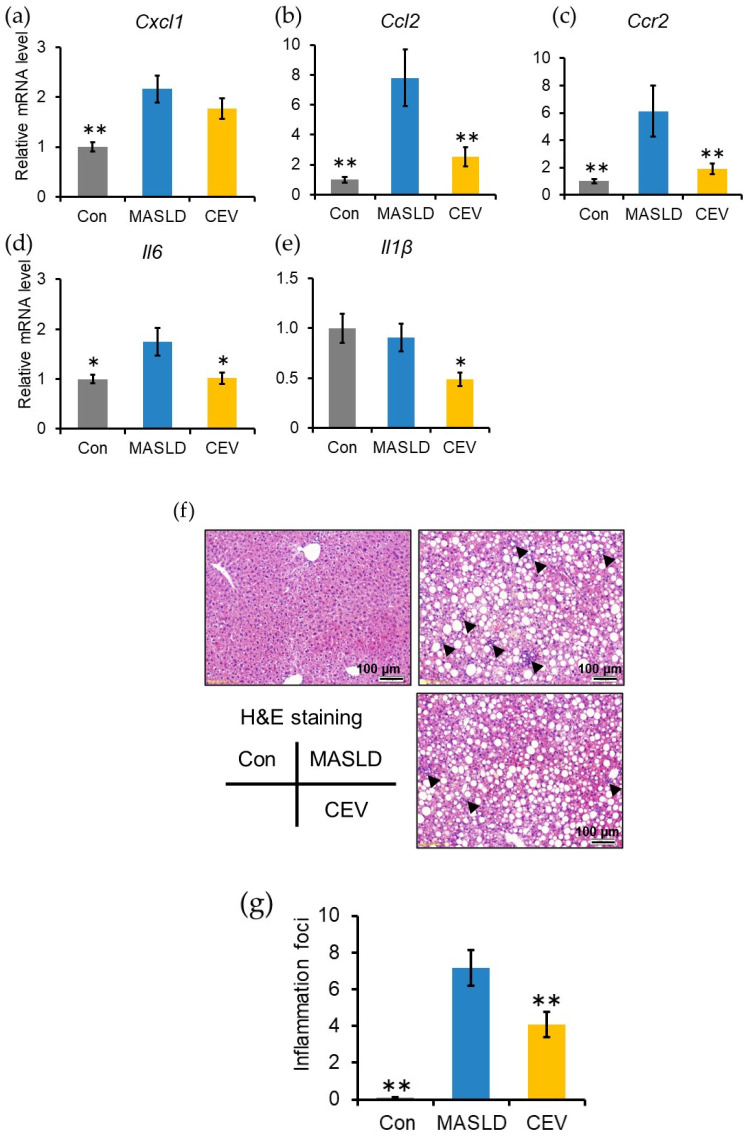
Effect of CEV administration on immune cell recruitment. (**a**–**e**) mRNA expression of inflammatory genes in the liver: (**a**) *Cxcl1*, (**b**) *Ccl2*, (**c**) *Ccr2*, (**d**) *Il6*, and (**e**) *Il1β*. (**f**) Representative images of hematoxylin and eosin (H&E)-stained liver sections; arrowheads indicate immune cell infiltration. (**g**) Number of lobular inflammatory foci per 100× field. Con, control; MASLD, metabolic dysfunction-associated steatotic liver disease; CEV, *Chlorella vulgaris*-derived extracellular vesicles. Data are expressed as mean ± SE (*n* = 5–8 per group). Statistical analyses were performed using one-way ANOVA followed by Dunnett’s test; * *p* < 0.05, ** *p* < 0.01 vs. MASLD group.

Lipid accumulation in hepatocytes triggers inflammation and activates hepatic stellate cells (HSCs) through chemokine and cytokine production [32]. Hepatic mRNA expression of inflammation-related genes was therefore analyzed (Figure 6a–e). *Cxcl1*, *Ccl2*, *Ccr2*, and *Il6* mRNA levels were significantly elevated in the MASLD group compared with the control group. CEV administration significantly reduced *Ccl2*, *Ccr2*, *Il6*, and *Il1b* expression levels.

To further assess inflammatory signaling at the protein level, we examined the expression of key NFκB pathway components (IKKα, IKKβ, p65, and phosphorylated p65) by Western blotting (Supplementary Figure S4). IKKα expression did not differ between the groups. IKKβ showed a non-significant trend toward higher expression in the MASLD group compared with the control group (*p* = 0.142). Total p65 tended to be lower in the CEV group than in the MASLD group (*p* = 0.101), and phosphorylated p65 tended to increase in the MASLD group and decrease following CEV treatment (*p* = 0.105 and *p* = 0.119, respectively). Although these differences did not reach statistical significance, the overall pattern was consistent with the mRNA-level suppression of inflammatory mediators.

Quantification of the lobular inflammatory foci, reflecting immune cell infiltration, revealed a significant increase in the MASLD group, whereas the CEV group showed a significant reduction (Figure 6f,g). These results collectively indicate that CEVs reduce inflammatory gene expression and immune cell recruitment, accompanied by protein-level changes consistent with attenuated NFκB pathway activation.

## 3. Discussion

**Figure 7:**
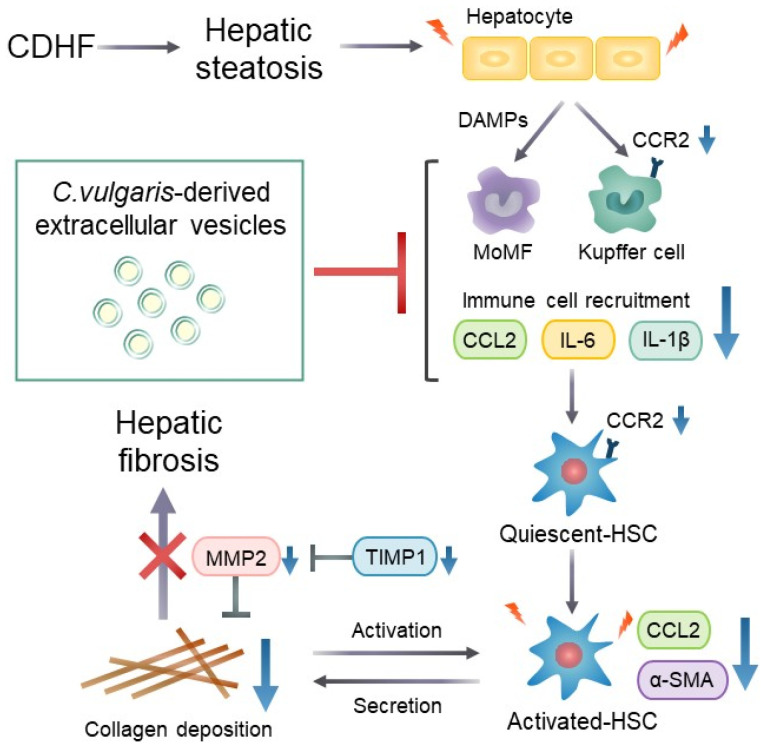
Possible mechanism by which CEVs suppress liver fibrosis in MASLD model.

In this study, we isolated extracellular vesicles derived from *Chlorella vulgaris* (CEVs) and evaluated their physiological effects in a CDHF-induced MASLD mouse model. Our findings demonstrate that oral administration of CEVs suppresses early inflammatory responses associated with hepatic steatosis and effectively attenuates subsequent excessive collagen deposition (Figure 7). Although interest in the biological activities of plant- and algae-derived EVs has been increasing, previous studies have focused primarily on in vitro effects or on EVs derived from terrestrial plants. To our knowledge, this is the first in vivo study to demonstrate the physiological functions of EVs derived from *C. vulgaris* and the first to show that microalgae-derived EVs can exert antifibrotic and anti-inflammatory effects following oral administration. These findings highlight a previously unrecognized therapeutic potential of microalgae-derived EVs as orally deliverable bioactive nanovesicles.

To characterize the physicochemical and morphological properties of the purified CEVs, we first performed nanoparticle tracking analysis and scanning electron microscopy imaging, which showed that their particle size ranged from approximately 100 to 250 nm (Figure 1) and spherical morphology (Supplementary Figure S1), consistent with the size range generally reported for plant-derived EVs (50 nm–1 μm) [33]. The mean particle size observed in this study (227.6 nm) was larger than that reported by Jin et al. (approximately 94 nm) [26], likely reflecting methodological differences, as their study employed sequential ultracentrifugation, whereas we used ultrafiltration to concentrate vesicles from the culture supernatant. Our isolation procedure followed the method of Morishita et al. [30], who successfully purified EVs from the microalga Pavlova using the same ultrafiltration-based approach and confirmed their vesicular morphology by electron microscopy. Their findings support the suitability of this method for isolating microalgae-derived EVs with high purity.

In addition to particle size, we further examined the protein profile of CEVs using proteinase K digestion followed by SDS-PAGE (Supplementary Figure S2). Several protein bands were susceptible to proteolysis in the absence of SDS, whereas others were protected unless SDS was added, suggesting the presence of both externally associated proteins and proteins shielded within vesicle structures. Although these findings do not exclude the possibility of co-isolated contaminants, they support the presence of protein components that are at least partially protected by a lipid membrane.

To explore the biological availability of orally administered CEVs, we analyzed plasma samples collected after administration and detected *C. vulgaris*-derived DNA at 1 h (Supplementary Figure S3). While this result does not directly demonstrate hepatic uptake, it indicates that at least a portion of the administered vesicles or their nucleic acid cargo can cross the gastrointestinal barrier and enter systemic circulation. Together with previous reports showing that plant-derived EVs from ginger [17], kiwi [34], and acerola [35] are absorbed through the intestine and accumulate in the liver, these findings support the biological plausibility that CEVs may reach hepatic tissue following oral delivery. Overall, the physicochemical and preliminary biodistribution data suggest that the CEVs isolated in this study exhibit characteristics broadly comparable with those of other plant-derived EVs and possess the potential to exert biological effects after oral administration.

To assess the protective effects of CEVs against MASLD-associated hepatic injury, we established a MASLD model by administering a CDHF diet to C57BL/6J mice. CDHF feeding is known to induce hepatic lipid accumulation while concurrently reducing body weight and whole-body adipose tissue because of nutrient-deficiency-driven alterations in peripheral energy metabolism [36]. In our previous study, administration of Gnetin C to CDHF-diet-induced MASLD mice significantly reduced hepatic fibrosis using eight animals per group [37]. These findings support the suitability of this model for evaluating antifibrotic effects. Consistent with these previous findings, mice in the MASLD group of the present study exhibited significantly lower body weight at the end of the experimental period (Figure 2a) compared with the control group. A reduction in peripheral adipose tissue generally leads to decreased production of adipose-derived factors such as free fatty acids and TNF α, both of which impair insulin action. Thus, the apparent improvement in insulin sensitivity observed in the MASLD group relative to controls may be attributable to these changes in adipose-derived signaling. In line with an earlier report demonstrating that CDHF feeding attenuates the rise in blood glucose following insulin administration [29], results from ITT in this study similarly showed significantly lower post-insulin blood glucose levels and reduced AUC in CDHF-fed mice (Figure 3b,c).

In the present study, CDHF feeding resulted in significant increases in liver weight (Figure 4a), total hepatic lipid content (Figure 4b), and hepatic TG levels (Figure 4c), along with a marked reduction in plasma lipid concentrations (Table 1). Histological examination revealed lipid droplet accumulation exceeding 5%, fulfilling one of the diagnostic criteria for hepatic steatosis (Figure 4f,g). Mechanistically, CDHF promotes hepatic lipid deposition by suppressing phosphatidylcholine biosynthesis and impairing VLDL assembly [38]. The observed reductions in plasma lipid levels are consistent with this mechanism, confirming the successful induction of hepatic steatosis.

During MASLD progression, excessive lipid accumulation induces lipotoxicity, which provokes recurrent inflammatory responses and ultimately culminates in fibrosis. In the MASLD group, fibrosis-related genes were significantly upregulated (Figure 5c–h), accompanied by pronounced collagen deposition within the extracellular matrix (ECM, Figure 5a,b). Hepatic cytokine and proinflammatory genes were also markedly upregulated (Figure 6a–e), and substantial immune cell infiltration was observed (Figure 6f,g). Collectively, these findings confirm that 12 weeks of CDHF feeding elicited pathological alterations characteristic of MASLD, including steatosis, lobular inflammation, and fibrosis.

No significant differences were detected between the MASLD and CEV groups in plasma lipid concentrations, liver weight, total hepatic lipid content, or hepatic TG and TC levels. These results indicate that CEV administration does not modulate hepatic steatosis in CDHF-fed MASLD model mice. In contrast, administration of *C. vulgaris* itself, rather than its secreted vesicles, has been reported to ameliorate hyperlipidemia and insulin resistance and to attenuate hepatic lipid accumulation in MASLD [24,25]. This discrepancy may reflect the involvement of bioactive constituents of *C. vulgaris* that are not incorporated into CEVs or, alternatively, differences in the pathological mechanisms underlying the respective disease models. Whereas Moradi et al. employed a high-fat-diet-induced MASLD model, the methionine-restricted high-fat diet used in the present study induces rapid, liver-specific lipid accumulation without promoting obesity or insulin resistance [36,39]. Consistent with these reports, CDHF feeding did not increase final body weight or non-fasting blood glucose levels relative to the control diet during the experimental period (Figure 3a).

The most significant finding of the present study is that CEV administration markedly attenuated hepatic fibrosis in CDHF-fed MASLD model mice (Figure 5a,b). Hepatic fibrosis is primarily driven by excessive production of ECM components by activated HSCs [40]. Quiescent HSCs store vitamin A; however, upon liver injury, they differentiate into myofibroblast-like cells in response to factors such as TGF-β [41]. In the current study, CDHF feeding significantly increased the mRNA expression of *Col1a1*, *Acta2*, and the HSC-derived ECM modulating factors *Mmp2* and *Timp1*, whereas CEV administration significantly downregulated all these markers, suggesting that CEVs suppress HSC activation.

Progression from hepatic steatosis to fibrosis is initiated by chronic inflammation resulting from sustained lipid overload. Hepatocytes damaged by lipotoxicity release damage-associated molecular patterns, which stimulate chemokine and proinflammatory cytokine production by monocyte-derived macrophages and resident Kupffer cells [42,43]. Kupffer cell-derived CCL2 and IL-6 are key inflammatory mediators that promote HSC activation [44,45]. CCR2, the receptor for CCL2, is expressed on both Kupffer cells and HSCs and facilitates macrophage recruitment to sites of injury [46]. In the present study, CDHF feeding significantly increased hepatic mRNA expression of *Ccl2*, *Ccr2*, and *Il6*, whereas CEV administration significantly reduced the expression of these genes, as well as *Il1β* (Figure 6b–e). Consistent with these transcriptional changes, Western blot analysis revealed trends toward reduced p65 and phosphorylated p65 expression in the CEV group compared with the MASLD group, although these differences did not reach statistical significance. These protein-level tendencies align with the observed suppression of inflammatory gene expression and suggest attenuation of NFκB pathway activation. Moreover, the number of lobular inflammatory foci, reflecting immune cell infiltration, was markedly reduced by CEV treatment (Figure 6f,g). Notably, *Ccr2* deficiency ameliorates CCl_4_-induced hepatic fibrosis, and CCR2 on Kupffer cells, rather than on HSCs, has been shown to mediate early inflammatory responses and immune cell infiltration [47]. The suppression of chemokine receptor gene expression, the reduction in lobular inflammation, and the protein-level trends indicating reduced NFκB activation collectively indicate that CEVs modulate early macrophage-driven inflammatory responses. These findings suggest that CEV administration mitigates ECM accumulation by suppressing early-stage hepatic inflammation associated with lipid overload and by preventing aberrant HSC activation, a key mechanism underlying MASLD progression.

Several previous studies support the concept that hepatic fibrosis can be attenuated even when steatosis remains unchanged, provided that steatosis-induced inflammation is effectively suppressed. Mridha et al. reported that pharmacological inhibition of NLRP3 in high-fat-diet-induced MASLD mice reduced hepatic inflammation and fibrosis, accompanied by decreased Il1β expression, despite no improvement in steatosis [48]. In a choline–methionine-deficient diet model, Deng et al. demonstrated that kinsenoside alleviated liver fibrosis by inhibiting the NF-κB/NLRP3 pathway [49], and Wang et al. showed that the ROR agonist nobiletin reduced hepatic inflammation and fibrosis through upregulation of KLF4 [50]. Collectively, these findings indicate that suppressing inflammation triggered by hepatic steatosis is sufficient to mitigate fibrosis independent of changes in lipid content. Our results are consistent with this concept, suggesting that CEVs attenuate fibrosis primarily through anti-inflammatory mechanisms rather than through direct effects on hepatic lipid accumulation.

Chronic hepatic inflammation is closely linked to the development of insulin resistance. Thus, inflammation driven by excessive hepatic lipid accumulation in MASLD is expected to impair IRS-mediated signaling pathways in the liver, ultimately reducing hepatic glucose uptake [51,52,53]. In the present study, CEV supplementation significantly lowered fasting glucose levels and reduced blood glucose concentrations following insulin administration (Figure 3b), indicating enhanced insulin sensitivity. Moreover, although CDHF feeding markedly increased hepatic *Il6* expression, CEV administration restored *Il6* levels to those observed in the control group (Figure 6d). Collectively, these findings suggest that CEVs may enhance insulin signaling by suppressing hepatic inflammation, thereby improving hepatic glucose uptake and systemic insulin sensitivity.

Macrophages are highly specialized phagocytic cells that play central roles in innate immune defense [54]. Previous studies have demonstrated that plant-derived EVs (e.g., from grapes and ginger) are taken up by macrophages and inhibit chemokine production and inflammasome activation [55,56]. The liver, which receives gut-derived molecules via the portal circulation, provides an environment in which resident Kupffer cells efficiently capture such particles. Indeed, grapefruit-derived EVs have been shown to localize to Kupffer cells following oral administration in mice [17]. These observations raise the possibility that orally administered CEVs are absorbed through the intestine and subsequently act on Kupffer cells to modulate inflammatory responses.

Our study suggests that CEVs may exert anti-inflammatory effects in the liver by downregulating several cytokines and chemokines. In addition to hepatic inflammation, plant-derived EVs have been reported to attenuate neuroinflammation, vascular inflammation, and acute lung injury [57]. Furthermore, previous studies have shown associations between increased spleen volume and MASLD [58], as well as links between splenic immune activation and metabolic liver injury [59]. Given that CEV-derived DNA was detectable in the circulation, it is possible that CEVs may also contribute to the suppression of inflammatory responses in the spleen, in addition to their effects in the liver. Further studies will be required to elucidate these mechanisms in more detail.

Plant- and algae-derived EVs are increasingly recognized as nanocarriers of bioactive molecules that are protected from intestinal enzymatic degradation and resistant to digestive conditions [14]. *C. vulgaris* contains several anti-inflammatory and antioxidant phytochemicals, including lutein, zeaxanthin, and chlorophylls [20,60,61]. Similar to turmeric-derived EVs, which are enriched in curcuminoids [62], CEVs may encapsulate carotenoids or other phytochemicals that contribute to their anti-inflammatory effects. Indeed, extracellular vesicles derived from prickly pear fruit juice have been shown to exert potent antioxidant effects in human skin fibroblasts, reducing intracellular reactive oxygen species (ROS) levels and promoting reparative processes [63]. Similarly, tea leaf-derived extracellular vesicles suppress hepatic stellate cell migration, and their cargo miR-44 mediates antifibrotic activity through downregulation of Smad2/3, ultimately attenuating carbon tetrachloride-induced liver fibrosis [64]. These findings support the concept that extracellular vesicles can function as nanovectors carrying antioxidative or antifibrotic molecules. Moreover, previous studies have demonstrated that arachidonic acid and docosahexaenoic acid—lipid components of EV membranes—ameliorate CCl_4_-induced liver injury in mice by downregulating chemokine receptors [65], suggesting that EVs may exert biological regulatory functions not only through their internal cargos but also via their intrinsic membrane lipids.

In addition to these phytochemicals, plant- and algae-derived EVs are known to carry diverse classes of bioactive molecules, including proteins, lipids, microRNAs, and DNA fragments. Given this molecular heterogeneity, and because our analyses did not allow us to isolate the effects of individual cargos, it is likely that multiple components of CEVs, rather than a single dominant molecule, contribute collectively to their anti-inflammatory and antifibrotic actions. In addition, previous reports have shown that arachidonic acid and docosahexaenoic acid, which are lipid components of EV membranes, can ameliorate CCl_4_–induced liver injury in mice by downregulating chemokine receptors [65]. The specific components within the CEVs used in this study that contributed to the antifibrotic effects remain unclear. Comprehensive profiling of the functional molecules contained within CEVs will therefore be essential for identifying the key factors responsible for their anti-inflammatory and antifibrotic activities in MASLD. Furthermore, the CDHF model is widely used for inducing steatohepatitis and fibrosis within a short timeframe, making it suitable for evaluating hepatic inflammation and fibrogenesis. However, its translational relevance to systemic metabolic dysfunction is limited. Our findings should be interpreted as liver-specific effects within this model, and it should be noted that future studies using metabolically relevant models (e.g., Western diet, high-fat diet) will be necessary to evaluate broader clinical applicability.

## 4. Materials and Methods

### 4.1. EV Isolation from C. vulgaris and Characterization

*C*. *vulgaris* was cultured in BG-11 medium at room temperature under a 12 h light/dark cycle. The culture supernatant was passed through a 0.22 μm filter; CEVs were concentrated tenfold by ultrafiltration (200K MWCO; ADVANTEC Ltd., Tokyo, Japan), sterilized by passage through a 0.22 μm filter, and stored at 4 °C until use [30]. The EV particle size and concentration were determined using a NanoSight LM10 (Malvern Panalytical Ltd., Malvern, UK).

### 4.2. Animals

All animal experimental protocols were approved by the Animal Ethics Committee of Tohoku University (approval number: 2025-AgA-004-02). Male C57BL/6J mice (7 weeks old) were purchased from CLEA Japan Inc. (Tokyo, Japan) and maintained under specific pathogen-free conditions with controlled temperature (23 ± 2 °C), humidity (55 ± 10%), and a 12 h light/dark cycle. Mice were randomly assigned into three groups (*n* = 8 per group) based on body weight and blood glucose level: Control, MASLD, and CEV. The Control group was fed the control diet (10% calories from fat; A06071314M, Research Diet Inc., New Brunswick, NJ, USA), whereas the MASLD and CEV groups were fed a methionine- and choline-deficient high-fat (CDHF) diet (0.2% methionine, 46% calories from fat; A06071318M, Research Diet Inc.) with free access to water. The composition of the diet is provided in Table S1. The CEV group received oral administration of *C. vulgaris*-derived EV twice weekly for 12 weeks (200 μL containing 1.63 to 3.45 × 10^11^ particles per dose), while the Control and MASLD groups were administered an equal volume of saline. Body weight and food intake were recorded weekly throughout the experimental period. After 12 weeks, mice were fasted for 5 h and sacrificed. Blood samples were collected from the heart under isoflurane anesthesia and centrifuged at 4 °C and 2000× *g* for 15 min; the obtained plasma was stored at −20 °C for subsequent analysis. Liver and epididymal white adipose tissue (eWAT) were collected and either stored at −60 °C or fixed in 10% neutral buffered formalin.

### 4.3. Insulin Tolerance Assessment

Blood glucose was measured at weeks 0, 6, and 12. Non-fasting blood was collected from the tail vein and analyzed using Nova StatStrip Express 900 (Siemens Healthcare, Co., Tokyo, Japan). At week 10, an insulin tolerance test (ITT) was performed. Mice were fasted for 3 h and injected intraperitoneally with inulin (Humulin R; Eli Lilly Japan K.K., Kobe, Japan) at 0.75 U/kg body weight; blood glucose was evaluated at 0, 30, 60, 90, and 120 min after injection.

### 4.4. Blood Biochemical Examination

Liver function markers, including alanine aminotransferase (ALT), aspartate aminotransferase (AST), alkaline phosphatase (ALP), lactate dehydrogenase (LDH), leucine aminopeptidase (LAP), cholinesterase (ChE), total bilirubin (T-BIL), and total bile acid (TBA), were measured. Plasma lipid profiles, including plasma triglycerides (TG), total cholesterol (TC), free cholesterol (FC), esterified cholesterol (EC), non-esterified fatty acid (NEFA), low-density lipoprotein cholesterol (LDL-C), and high-density lipoprotein cholesterol (HDL-C), were also determined. Nutritional status was assessed by measuring plasma total protein (TP) and albumin (ALB). All analyses were outsourced to Oriental Yeast Co., Ltd. (Siga, Japan).

### 4.5. Quantification of Hepatic Lipid Accumulation

Hepatic total lipids were extracted according to Folch’s method. Briefly, approximately 250 mg of liver tissue was homogenized in a glass tube with methanol–chloroform (1:2, *v*/*v*) using a polytron homogenizer (PT2500E, Kinematica AG, Malters, Switzerland). After centrifugation at 900× *g* for 10 min, the supernatant was collected and mixed with 0.05% H_2_SO_4_. The mixture was vortexed and centrifuged at 900× *g* for 10 min. The lower organic layer was evaporated to dryness, and the isolated lipids were dissolved in distilled water. TG, TC, and NEFA levels were quantified using the commercial kits (#291-94501, #293-93601, #299-94301, FUJIFILM Wako Pure Chemical Co., Osaka, Japan) according to the manufacturer’s instructions.

### 4.6. Liver Histopathological Analysis

Liver tissues were fixed in 10% neutral formalin, dehydrated with graded ethanol, and embedded in paraffin. Sections (4 μm) were prepared for hematoxylin and eosin (H&E) staining and picrosirius red staining. Images were acquired using an Olympus IX81S1F-3 (Olympus, Tokyo, Japan) with a 10× objective lens. H&E-stained sections were analyzed with ImageJ software (version 1.37c, Wayne Rasband, National Institute of Health, Bethesda, MD, USA) to quantify the steatosis area and the average size of the lipid droplet size. Picrosirius red-stained sections were similarly analyzed to quantify fibrotic area based on collagen deposition visualized in red.

### 4.7. RNA Extraction and Quantitative Reverse Transcription Polymerase Chain Reaction

Liver samples for RNA extraction were subsequently preserved in RNAlater. qRT-PCR analysis was performed as previously described [37]. The total RNA was extracted using ISOGEN (Nippon Gene Co., Ltd., Tokyo, Japan). RNA purity and concentration were determined with a Nanodrop spectrophotometer (Thermo Fisher Scientific K.K., Tokyo, Japan) by calculating the absorbance ratio at 260/280 nm. cDNA was synthesized by mixing purified RNA with 50 ng/μL oligo d(T) and 1 mM of dNTPs and incubating the mixture for 5 min at 65 °C, followed by reverse transcription in RT buffer (50 mM of Tris-HCl at pH 8.3, 75 mM of KCl, 3 mM MgCl_2_, and 5 mM of dithiothreitol) containing 50 U of SuperScript III reverse transcriptase (Invitrogen, Carlsbad, CA, USA) and 20 U of RNaseOUT RNase inhibitor (Invitrogen) at 50 °C for 60 min. qPCR was performed using THUNDERBIRD Next SYBR qPCR Mix (TOYOBO, Osaka, Japan) and gene-specific primers (Table S2) on a Bio-Rad CFX Connect real-time PCR detection system (Bio-Rad laboratories Inc., Hercules, CA, USA). Eukaryotic elongation factor (Eef1α1) was used as the reference gene.

### 4.8. Statistics

Results are expressed as mean ± standard error (SE). Statistical analyses were performed using SigmaPlot version 12.5 (Systat Software Inc., San Jose, CA, USA). Data were analyzed using one-way ANOVA followed by Dunnett’s test (vs. MASLD group). Body weight and food intake were analyzed using two-way repeated-measures ANOVA followed by Dunnett’s test (vs. MASLD group). Differences were considered statistically significant at *p* < 0.05.

## 5. Conclusions

This study demonstrates for the first time that oral administration of *C. vulgaris*-derived extracellular vesicles (CEVs) inhibits the progression of liver fibrosis in a murine model of MASLD. These findings strongly suggest that CEVs constitute a novel nutraceutical candidate for preventing the progression of MASLD.

## Figures and Tables

**Table 1:** Plasma biochemical parameters.

	Control	MASLD	CEV
Plasma Liver Injury Marker			
AST (IU/L)	82 ± 15	121 ± 16	133 ± 8
ALT (IU/L)	18 ± 2 **	118 ± 10	132 ± 8
ALP (IU/L)	59 ± 2 *	67 ± 1	72 ± 3
LDH (IU/L)	207 ± 30	291 ± 31	342 ± 16
LAP (IU/L)	35 ± 1 **	59 ± 2	60 ± 3
ChE (IU/L)	19 ± 1	24 ± 1	24 ± 1
T-BIL (mg/dL)	0.06 ± 0.01	0.08 ± 0.01	0.09 ± 0.01
TBA (μmol/L)	1.5 ± 0.2	3.4 ± 0.5	5.5 ± 1.0
Plasma Lipids			
TG (mg/dL)	36 ± 4 *	24 ± 1	22 ± 1
TC (mg/dL)	113 ± 5 **	77 ± 3	76 ± 4
FC (mg/dL)	30 ± 1 *	22 ± 1	21 ± 1
CE (mg/dL)	83 ± 4 *	55 ± 2	54 ± 3
E/T (%)	73.6 ± 0.4	72.0 ± 0.6	71.8 ± 0.6
NEFA (μEq/L)	783 ± 62 **	542 ± 21	636 ± 40
LDL-C (mg/dL)	5.1 ± 0.4 **	2.4 ± 0.2	2.0 ± 0.3
HDL-C (mg/dL)	62 ± 2 **	44 ± 1	42 ± 3
Nutritional Status Index in Plasma			
TP (g/dL)	4.8 ± 0.1	4.7 ± 0.1	4.6 ± 0.1
ALB (g/dL)	3.06 ± 0.04	2.99 ± 0.02	2.96 ± 0.08

AST, aspartate aminotransferase; ALT, alanine aminotransferase; ALP, alkaline phosphatase; LDH, lactate dehydrogenase; LAP, leucine aminopeptidase; ChE, cholinesterase; T-BIL, total bilirubin; TBA, total bile acid; TG, triglyceride; TC, total cholesterol; FC, free cholesterol; CE, esterified cholesterol; E/T, the ratio of cholesterol ester to total cholesterol; NEFA, non-esterified fatty acid; LDL-C, low-density lipoprotein cholesterol; HDL-C, high-density lipoprotein cholesterol; TP, total protein; ALB, albumin. MASLD, metabolic dysfunction-associated steatotic liver disease; CEV, *Chlorella vulgaris*-derived extracellular vesicles. Data are expressed as mean ± SE (*n* = 8 per group). Statistical analyses were performed using one-way ANOVA followed by Dunnett’s test; * *p* < 0.05, ** *p* < 0.01 vs. MASLD group.

## Data Availability

All data generated or analyzed in this study are provided within the article. Additional information is available from the corresponding author upon request.

## Supplementary Materials

The following supporting information can be downloaded at https://www.mdpi.com/article/10.3390/ijms27093735/s1. References [66,67,68] are cited in Supplementary Materials.
