# Supplementary material for: Extracellular Vesicle from Chlorella vulgaris Alleviates Hepatic Fibrosis in a Mouse Model of Metabolic Dysfunction-Associated Steatotic Liver Disease Through Modulation of Inflammatory Signaling
Source: Int J Mol Sci. 2026 Apr 22;27(9):3735. doi: 10.3390/ijms27093735 (PMC13163729; doi:10.3390/ijms27093735)
Supplement: Supplementary file 1 [file ijms-27-03735-s001.zip › ijms-4198981-supplementary.pdf]

Supplementary Methods

Scanning Electron Microscopy analysis

CEVs were fixed with 2.5% glutaraldehyde at room temperature for 1 h and then dehydrated sequentially with 30%, 50%, and 70% ethanol. After incubation in 1% phosphotungstic acid prepared in 70% ethanol for 30 min, the samples were washed three times with 70% ethanol and further dehydrated with 100% ethanol. Ultrafiltration (100k MWCO, Microcon-100, Takara Bio Inc., Kusatsu, Japan) was used for solution exchange. The CEV particles were then observed using a scanning electron microscope (Miniscope TM4000; Hitachi High-Technologies, Tokyo, Japan).

Proteinase K protection assay

CEV solutions were incubated for 30 min in the presence or absence of 0.2% SDS with a proteinase K reaction buffer (100 mM Tris-HCl, 5 mM EDTA, 200 mM NaCl, 1 μg/mL proteinase K, pH 8.0). Proteinase K–treated and untreated CEVs were then denatured with SDS and separated on an 12.5% polyacrylamide gel. Proteins in the polyacrylamide gel were stained using the Silver Stain 2 Kit Wako (291-50301, FUJIFILM Wako Pure Chemical Corporation, Osaka, Japan) and scanned with a ChemiDoc Touch imaging system (Bio-Rad Laboratories, Inc., Tokyo, Japan).

Detection of C. vulgaris-derived DNA in mouse plasma after oral administration of CEVs

Blood samples were collected from C57BL/6 mice at 1 hour or 6 hours after oral administration of 200 μL of CEVs, and plasma was obtained by centrifugation (4 °C and 2,000 × g for 15 min). DNA was extracted from the plasma using the phenol–chloroform method. PCR amplification was performed using *C. vulgaris* specific primers [3] (5′-TACGTGCGTAAATCCCGACT-3′ and 5′-ACCCGAAATCCAACTACGAG-3′) and PrimeSTAR HS DNA polymerase (Takara Bio Inc.). The PCR products were separated on a 1.5% agarose gel by electrophoresis at 150 V for 40 min. The gel was stained with GelRed (Biotium, Inc., Fremont, CA, USA) and visualized using a UV transilluminator.

Western blot analysis

Liver tissue (≈100 mg) was homogenized in 1 mL PBS containing cOmplete protease inhibitor cocktail (Merck, Darmstadt, Germany) and PhosSTOP (Merck). Homogenates were centrifuged at 14,000× g for 10 min at 4 °C, and protein concentrations in the supernatants were determined using the Protein Assay Dye Reagent (Bio-Rad Laboratories, Inc.).

Protein samples (20 μg/15 μL) were loaded (10 μL/lane) onto SuperSep Ace 12.5% gels (FUJIFILM Wako Pure Chemical, Osaka, Japan) and separated by SDS–PAGE (150 V, 80 min). Proteins were transferred to Immobilon-P membranes (Millipore, Burlington, MA, USA) using a semi-dry system (300 V, 30 min). Membranes were blocked in TBS-T containing 5% BSA for 1 h at room temperature and incubated overnight at 4 °C with primary antibodies for IKKα (1:1000, #2682, Cell Signaling Technology, Danvers, MA, USA), IKKβ (1:1000, #2678, Cell Signaling Technology), p65 (1:1000, #8242, Cell Signaling Technology), phospho-p65 (1:1000, #3033, Cell Signaling Technology). After washing, membranes were incubated with HRP-conjugated anti-rabbit IgG (1:5000) for 1 h at room temperature.
Signals were developed using Immobilon Western reagents (Millipore) and detected with a ChemiDoc Imaging System (Bio-Rad Laboratories). Band intensities were quantified using Image Lab 6.1. GAPDH (1:10000, #97166, Cell Signaling Technology) served as a loading control, and target protein levels were normalized to GAPDH and expressed relative to the Control group.

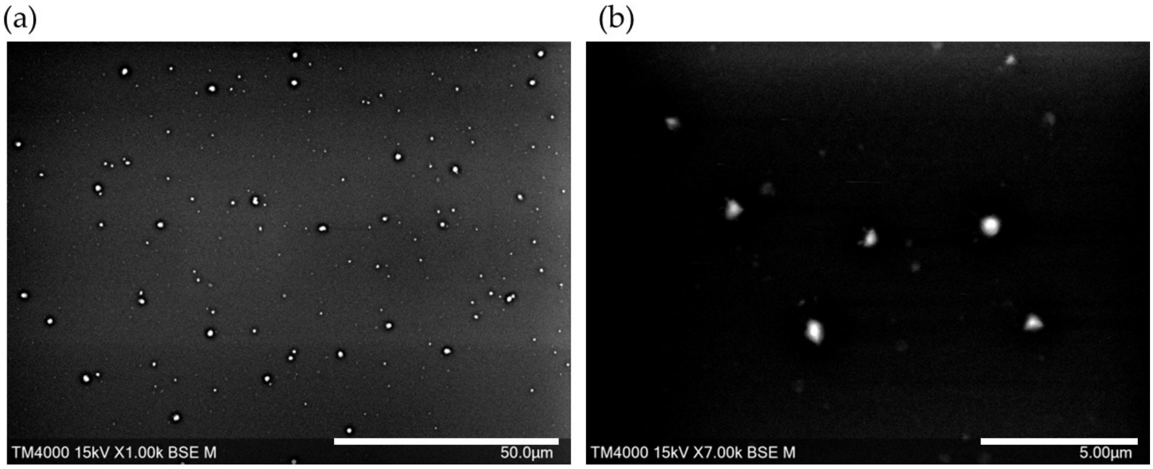

Supplementary Figure 1. Scanning electron microscopy of CEVs.
CEVs were fixed and observed using a scanning electron microscope. Representative images are shown at (a) 1,000× and (b) 7,000× magnification.

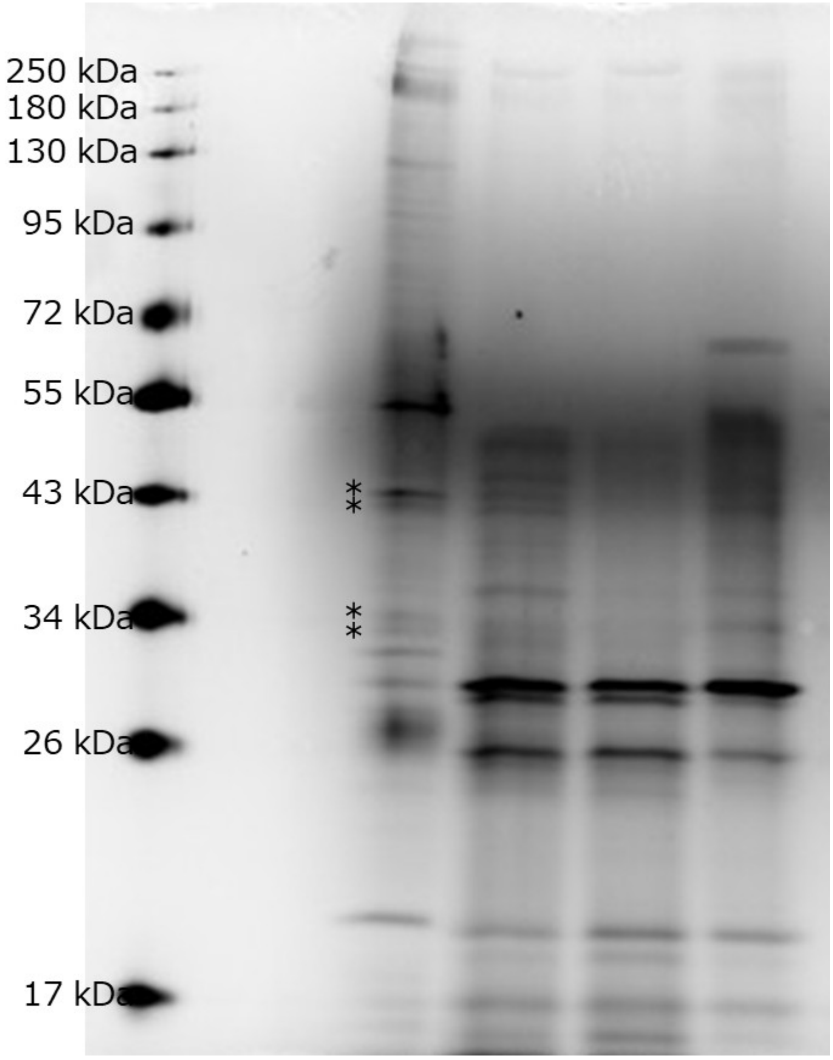

Supplementary Figure 2. Proteinase K protection assay.

CEVs or proteinase K–treated CEVs were separated on a 12.5% polyacrylamide gel and visualized by silver staining.

M: protein molecular weight marker (details)

1: untreated CEVs

2: CEVs treated with proteinase K in the absence of SDS

3: CEVs treated with proteinase K in the presence of SDS

4: proteinase K reaction buffer without CEVs

Bands marked with * indicate protein bands that were detectable in untreated CEVs and in CEVs treated with proteinase K in the absence of SDS, but disappeared upon proteinase K treatment in the presence of SDS.

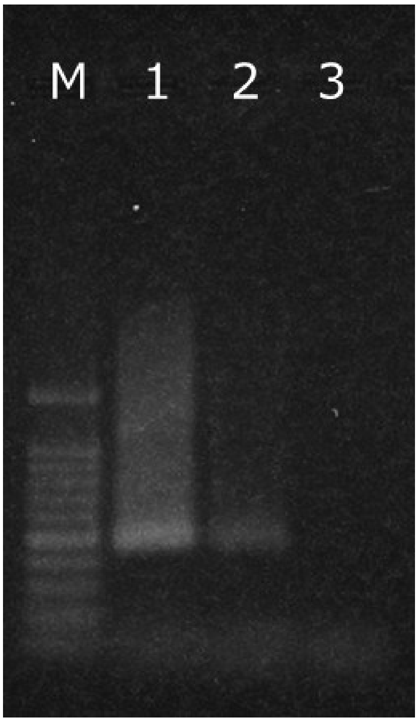

Supplementary Figure 3. Detection of *C. vulgaris*-derived DNA in mouse plasma after oral administration of CEVs

Agarose gel electrophoresis of PCR products amplified using C. vulgaris–specific primers. M: 100 bp DNA ladder. Lane 1: positive control (PCR product amplified from DNA extracted from CEVs). Lanes 2–3: PCR products amplified from plasma collected 1 h (lane 2) and 6 h (lane 3) after oral administration of CEVs. PCR products were separated on a 1.5% agarose gel at 150 V for 40 min, stained with GelRed, and visualized under a UV transilluminator.

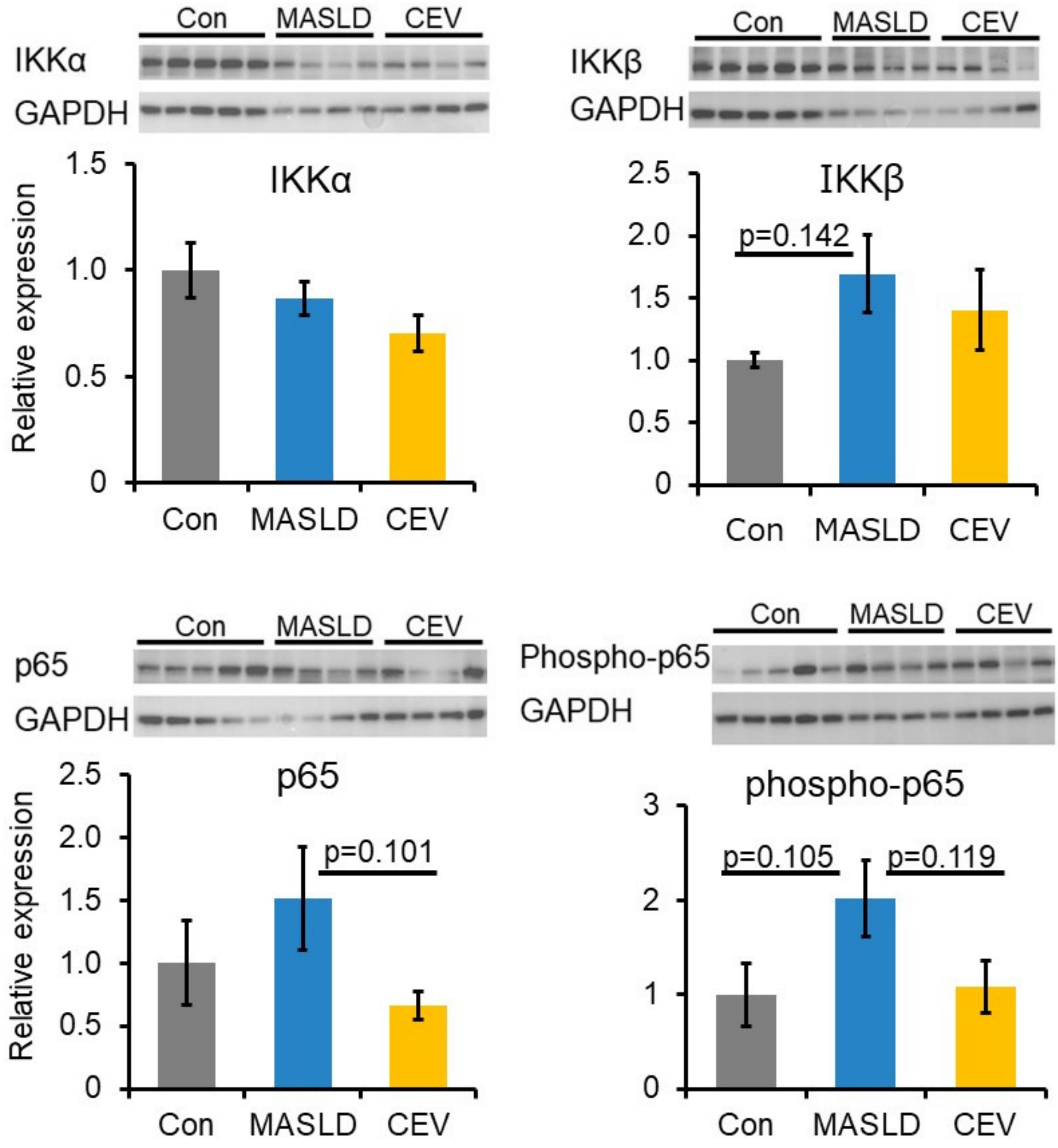

Supplementary Figure 4. Western blot analysis of NF-κB pathway–related proteins in liver tissue.

Representative Western blot images showing the expression levels of IKKα, IKKβ, p65, and phosphorylated p65 in liver samples. Equal amounts of total protein were subjected to SDS–PAGE and immunoblotted with specific antibodies against each target protein. Band intensities were quantified by densitometry and normalized by GAPDH expression. Bar graphs show the relative protein expression levels calculated. Con, control; MASLD, metabolic dysfunction-associated steatotic liver disease; CEV, Chlorella vulgaris-derived extracellular vesicles. Data are expressed as mean ± SE (n = 8 per group). Statistical analyses were performed using one-way ANOVA followed by Dunnett's post hoc test.

Table S1: Composition of experimental diets used in the present study.

	Control	CDHF
**Research Diets Inc. ID**	**A06071314M**	**A06071318M**
Ingredient (g)		
L-Glutamic acid	38.20	38.20
L-Proline	17.80	17.80
L-Leucine	15.80	15.80
L-Lysine, HCL	13.20	13.20
L-Aspartic Acid	12.10	12.10
L-Serine	10.00	10.00
L-Valine	9.30	9.30
L-Tyrosine	9.20	9.20
L-Phenylalanine	8.40	8.40
L-Isoleucine	7.60	7.60
L-Threonine	7.20	7.20
L-Arginine	6.00	6.00
L-Alanine	5.10	5.10
**L-Methionine**	**5.10**	**1.70**
L-Histidine	4.60	4.60
L-Cystine	4.20	4.20
Glycine	3.00	3.00
L-Tryptophan	2.10	2.10
Corn Starch	502.00	76.20
Maltodextrin, Lodex 10	130.10	100.00
Sucrose	72.80	176.80
Cellulose, FCC20	50.00	50.00
Soybean Oil	25.00	25.00
Lard	20.00	177.50
Mineral Mix S10026B	50.00	50.00
Sodium Bicarbonate	7.50	7.50
**Choline Bitartrate**	**2.00**	**0**
Vitamin Mix V10001C	1.00	1.00
FD&C #5 Yellow Dye	0.04	0.01
FD&C #1 Blue Dye	0.01	0.04
Total	1039.35	839.55
% (w/w)		
Protein	17.21	20.90
Carbohydrate	67.82	42.04
Fat	4.33	24.12
Kcal%		
Protein	18.16	17.83
Carbohydrate	71.56	35.87
Fat	10.28	46.30
Calories per 100 g (kcal)	379.09	468.84

Table S2: Nucleotide sequence for gene-specific primer.

Gene name	Forward primer (5′-3′)	Reverse primer (5′-3′)
Eef1α1	GATGGCCCCAAATTCTTGAAG	GGACCATGTCAATGGCAG
Col1α1	GTACATCAGCCCGAACCCCA	GGTGGACATTAGGCGCAGGA
Acta2	CAGATGTGGATCAGCAAACAGGA	GACTTAGAAGCATTTGCGGTGG
Mmp1α	ACTACAACTGACAACCCAAGAAAG	CCTGTTCCTGTTTTCAGAGCCA
Mmp2	GATAACCTGGATGCCGTCGTG	GGTGTGCAGCGATGAAGATGATA
Timp1	GGAACGGAAATTTGCACATCAG	CTGATCCGTCCACAAACAGTGAG
Tgfb1	AAGGAGACGGAATACAGGGCTT	CTGTCACAAGAGCAGTGAGCG
Cxcl1	TTGTGCGAAAAGAAGTGCAG	TACAAACACAGCCTCCCACA
Ccl2	GTTGGCTCAGCCAGATGCA	AGCCTACTCATTGGGATCATCTTG
Ccr2	GTGGTCTGGCTTTGCTACCAC	ATTAAAATGAAACTGTTAGGCTATCTGAAG
Il6	AGAGGAGACTTCACAGAGGATGC	AATCAGAATTGCCATTGCACAAC
Il1β	TCCAGGATGAGGACATGAGCAC	GAACGTCACACACCAGCAGGTTA

References

1. Hirose T, Ito H, Endo A, Sato S, Takahashi C, Kaburaki T, Yano K, Ishikawa R, Kamada A, Oba-Yabana I, Satoh M, Morozumi K, Kaiho Y, Nakamura Y, Kamijo K, Yumura W, Mori T. Reliable Detection of SGLT2 Protein by Knockout-Based Antibody Characterization. *Hypertension*. (2026) doi: 10.1161/HYPERTENSIONAHA.125.26135.
2. Matsuki T, Hirose T, Ohsaki Y, Shimada S, Endo A, Ito H, Takahashi C, Yamakoshi S, Oba-Yabana I, Anan G, Kato T, Tajima R, Nakayama S, Kimura T, Nakamura H, Tani J, Takahashi K, Kure S, Mori T. Inhibition of platelet-derived growth factor pathway suppresses tubulointerstitial injury in renal congestion. *J Hypertens*. 40(10):1935−1949. (2022) doi: 10.1097/HJH.0000000000003191.
3. Premina S, Niren AS, Sundaralingam R, Murugan N, Sharanya TV, Standardization and application of PCR targeting Chlorella species isolated from environmental samples. *Int J Pharm Pharm Sci*, 13(7), 28−31. (2021)
